# Producing Aerogels from Rice Straw Cellulose Obtained by a Green Method and Its Starch Blending

**DOI:** 10.3390/polym17081103

**Published:** 2025-04-18

**Authors:** Pedro A. V. Freitas, Paula Alonso Collado, Chelo González-Martínez, Amparo Chiralt

**Affiliations:** Institute of Food Engineering FoodUPV, Universitat Politècnica de València, 46022 Valencia, Spain; palocol@etsiamn.upv.es (P.A.C.); cgonza@tal.upv.es (C.G.-M.); dchiralt@tal.upv.es (A.C.)

**Keywords:** agro-industrial waste, cellulose fibres, mechanical properties, blended aerogels, green extraction methods

## Abstract

Cellulose and starch–cellulose composite aerogels were obtained using green cellulose from rice straw (RS) purified with a more environmentally friendly process. Pure starch aerogels were also obtained for comparison purposes. The effect of the aerogel cross-linking with polyamideamine-epichlorohydrin (PAE) was also analysed. The properties of the cellulose aerogels were in the range of those reported using other RS cellulose fibres with similar compositions. Blending with starch implied a decrease in the liquid water absorption capacity but an increase in the mechanical strength, flexibility, and oil absorption capacity, compared to pure cellulose aerogels. Cross-linking with PAE promoted the water adsorption capacity of all aerogels and the oil absorption capacity and mechanical strength of cellulose aerogels. However, PAE did not benefit the strength and oil absorption capacity of aerogels containing starch due to their specific interactions that negatively affect the aerogel structure. Therefore, it was possible to obtain cellulose and cellulose–starch composite aerogels from RS green cellulose with modulated properties for different applications.

## 1. Introduction

Valorisation of agri-food waste is a crucial practice of the circular economy, which focuses on obtaining added-value materials and optimises the use of renewable resources. Rice is a cereal crop that provides energy and protein for half of the world’s population. Its production has increased almost 40-fold since 1961, and global rice production in 2023/24 was estimated to reach a record of about 800 million tonnes, being 1.5% above the 2022/23 estimation [1]. Rice straw (RS) is a post-harvest by-product of rice, generating 5–6 tonnes of straw per hectare of rice (1.5 kg of RS/kg of rice grain), with a global estimation of almost 740–1111 million tonnes per year [2,3]. Traditionally, RS is burned in the field, which causes health problems in the areas surrounding the crop, as well as environmental problems due to emissions of carbon dioxide (CO_2_), dioxins, methane (CH_4_), and other gases [4]. In this sense, alternative management systems are being proposed, such as the removal of straw from rice fields to give it a new use, and its valorisation, including energy generation, compost, paper, levulinic acid for plastics, additives, and herbicides, among others [2]. Given its lignocellulosic composition, RS can be considered as a renewable source of cellulose, which could be applied in developing advanced biodegradable materials for food, pharmaceutical, or cosmetic uses.

To obtain cellulose, the hemicellulose, lignin, and other plant components must be removed, thus improving the crystallinity and thermal stability of the cellulosic material [5,6]. Nevertheless, traditional cellulose purification methods, such as alkaline treatment applying sodium or potassium hydroxide solutions, combined with a bleaching step using chlorinated solvents (hypochlorite, chlorite), are associated with environmental problems, due to the excessive use of chemicals that generate large quantities of effluents to be treated and disposed of, as well as the large quantities of water used after each purification step. In this sense, faster, scalable, chemical-free, and cost-effective methods have been proposed, using subcritical water extraction (SWE) and bleaching with greener solvents, such as hydrogen peroxide [7]. The high extractive power of water under subcritical conditions [8,9] allows for removing a part of lignin and hemicellulose of the biomass, favouring the subsequent delignification step. In this step, the use of hydrogen peroxide, through the action of the perhydroxyl anions (HOO^−^), allows for oxidising the lignin aromatic rings [10,11] without the formation of eco-toxic chlorinated derivatives in a more environmentally friendly process [12,13]. Nevertheless, the obtaining process affects the degree of purity and functionality of cellulose for a determined application, including paper and board production or advanced nanomaterials such as aerogels [14,15].

Aerogels are derived from hydrogels, whose liquid phase is replaced by gas through a solvent elimination process, generating a highly porous and ultra-light 3D structure containing about 90% air in its volume [16]. Their low density (0.003–0.500 g·cm^−3^), high porosity (80–99.8%), large specific surface area (100–1600 m^2^·g^−1^), and high surface chemical activity are some of the unique characteristics that make aerogels stand out among other solid materials [17,18]. Aerogels based on cellulose have a series of advantages, such as their high compressive strength (5.2 kPa–16.7 MPa) and their biodegradability. These have a great number of applications, such as water/oil absorption and separation, thermal insulation, biomedical materials, metal nanoparticle/metal oxide carriers, or carbon aerogel preparation. RS cellulose has been used to produce aerogels with different properties depending on the cellulose purification method [16,19,20,21]. Freitas et al. [21] observed clear differences in the aerogel properties depending on the RS cellulose purity, which were achieved using different extraction methods, combined with a sodium chlorite bleaching. However, no previous studies have reported the properties of aerogels prepared with green cellulose from RS. This can be produced using a previously optimised, more ecological method, combining subcritical water extraction and a bleaching process with hydrogen peroxide that does not produce chlorinate toxic residues [7]. This green cellulose could also be combined with other hydrocolloids, such as starch, to produce tuned aerogels for different uses.

To favour the water resistance of hydrophilic cellulose aerogels, polyamideamine-epichlorohydrin (PAE) has been previously used [16]. This is a water-soluble aliphatic polyimide resin containing a high number of active groups, such as azetidinium and alkyl groups. These enable the cross-linking of cellulose fibres by forming ester bonds with the carboxyl groups of the residual hemicellulose and the azetidinium group, thus strengthening the three-dimensional structure of the aerogel [22,23,24]. In addition to cross-linking with carboxyl groups, PAE gives rise to a self-cross-linking reaction that also contributes to the strength of the material. Likewise, combining cellulose with other polysaccharides, such as starch, has also gained increasing interest in obtaining aerogels with potential uses in different areas [25,26,27,28]. Due to their characteristics, including non-allergenicity, non-toxicity, abundance, and low cost, starch aerogels are particularly attractive and suitable for food and nutrition-related uses [29]. Starch-based aerogels have some limitations due to their low strength, especially in wet conditions. However, reinforcing the starch matrix with cellulose may improve their properties, such as water and mechanical resistance [30]. Cellulose–starch composite aerogels have been recently studied, offering interesting properties for several uses [29,30,31,32,33,34]. Paulauskiene et al. [35] reported high values of compressive modulus and high oil absorption capacity for aerogels from recycled cellulose and potato starch.

In this aforementioned sense, the aim of this study was to produce and characterise the properties of RS green cellulose aerogels, obtained by SWE and delignification with hydrogen peroxide. The potential of cellulosic fibres to form blended aerogels with potato starch, with modulated properties, was also evaluated. Likewise, the effect of a cross-linking resin (PAE) on the properties of the obtained aerogels was also analysed.

## 2. Materials and Methods

### 2.1. Plant Materials and Chemicals

RS (*Oryza sativa* L.), variety *J. Sendra*, was supplied by “Banco de Paja” from L’Albufera paddy fields (Valencia, Spain). This material consisted of rachis of the panicle, leaf blades, sheath, and the stalk bulk, as harvested in the field. The obtained RS was vacuum-dried at 50 ± 2 °C and 0.8 mmbar for 16 h, milled using a mill machine (IKA, model M20, IKA Werke GmbH & Co. KG, Staufen, Germany) for 3 cycles of 90 s each, sieved to obtain particles < 0.5 mm, and then stored in a desiccator containing P_2_O_5_ (relative humidity (RH) of ~0%) until further use. The composition of the used RS, as previously reported [7], was 36% cellulose, 19% hemicellulose, 21% lignin, and 17% ashes.

Potato starch was supplied by Roquette Frères (Lestrem, France). Polyamideamine-epichlorohydrin (PAE) resin (solution at 12% wt.) was obtained from Solenis (Alpharetta, Georgia, EUA). Sodium hydroxide (NaOH), magnesium sulphate (MgSO_4_), glucose, arabinose, and potassium iodide (KI) were obtained from Sigma-Aldrich (St. Louis, MO, USA). D(+)-Xylose was purchased from Merck KGaA (Darmstadt, Germany). Hydrogen peroxide (H_2_O_2_, 30%), ethanol, sulphuric acid (H_2_SO_4_, 98%), sodium carbonate (Na_2_CO_3_, 99.5%), potassium nitrate (KNO_3_), potassium carbonate (K_2_CO_3_), potassium chloride (KCl), magnesium chloride (MgCl_2_), lithium chloride (LiCl), and phosphorus pentoxide (P_2_O_5_, 98.2%) were supplied by Panreac Química S. L.U. (Castellar del Vallés, Barcelona, Spain). Sodium chloride (NaCl) was supplied by Labbox España (Premià de Dalt, Barcelona, Spain). Sunflower oil was supplied by Riazor S.A. (Riachos, Portugal).

### 2.2. Obtaining RS Cellulosic Fraction

The cellulose from RS was obtained with a two-step process as described by Freitas et al. [7]. First, the ground RS was subjected to an SWE with an RS: distilled water ratio of 1:10 (*w*/*v*) using a pressure reactor (Model 1-T-A-P-CE, 5 L capacity, Amar Equipment PVT. LTD, Mumbai, India), operating at 180 °C, 11 bar and 150 rpm, for 30 min. Afterward, the plant dispersion was filtered through a qualitative filter (Filter lab, Cambridge, MA, USA, pore size < 0.5 mm), and the obtained insoluble residue was washed with water to remove the remaining soluble fraction, followed by drying at 35 °C for 48 h. This cellulose-enriched fraction was bleached to remove lignin and hemicellulose residues. For this, the RS lignocellulosic residue was treated with 4% (*v*/*v*) H_2_O_2_, at pH 12 (using NaOH) and 40 °C for 1 h. The fibres were then filtered and washed with distilled water to remove the residual bleaching solution. This procedure was repeated four times. The bleached solid fraction was dried (30 °C for 48 h), ground in a mill (Model M20, IKA Werke GmbH & Co. KG, Staufen IKA, Staufen, Germany) with 2 s pulses for 20 min to reduce the particle size and stored in a desiccator at 0% RH (P_2_O_5_) until further use. These fibres contain 86% cellulose, 1.5% hemicellulose, 3.4% lignin, and 6% ashes, as determined in a previous study [7] by using the NREL/TP-510-42618-2008 method. The lengths of the fibres were below 140 μm, with thickness values between 7 and 15 μm [7]. And major cumulative frequencies of lengths and thicknesses of below 200 μm and 5–15 μm, respectively.

### 2.3. Production of Aerogels

Cellulose aerogels were obtained by dispersing cellulose fibres at 0.5 and 1% wt. in distilled water, followed by homogenisation with a rotor-stator homogeniser (Model T25 digital ULTRA-TURRAX, IKA Werke GmbH & Co. KG, Staufen IKA, Staufen, Germany) at 10,000 rpm for 30 min. Afterward, the cellulose dispersions were sonicated for 20 min in an ultrasonic homogeniser with a high-intensity probe (Vibra Cell ™VCX750, Sonics & Material, Newtown, CT, USA), applying a frequency of 20 kHz, with 40% amplitude, and maintaining 25 °C by the sample immersion in an ice bath. These dispersions were used to obtain pure cellulose aerogels (C0.5 and C1). To obtain the starch aerogels (S1 and S2), aqueous dispersions of potato starch (1 and 2% wt.) were gelatinised at 90 °C and 200 rpm for 60 min. The composite starch–cellulose aerogels (S1-C0.5 and S2-C0.5) were prepared by incorporating the corresponding amount of potato starch into the sonicated cellulose dispersion (0.5% wt.), followed by heating at 90 °C and 200 rpm for 1 h. To obtain the PAE-cross-linked aerogels, the necessary amount of PAE liquid dispersion (initial concentration of 12% wt.) was added to each dispersion to achieve an 8% wt. of PAE with respect to the total starch and/or cellulose mass. The aerogels with PAE were named by adding P to the initial notation (C0.5-P, C1-P, S1-P, S2-P, S1-C0.5-P, and S2-C1-P).

To obtain the different aerogels, the aqueous dispersions (1.5 mL for each sample) were placed in cylindrical vials (15 × 30 mm) (Vidrafoc, Barcelona, Spain) and frozen at −40 °C for 24 h. Afterwards, the frozen samples were freeze-dried (Telstar, model LyoQuest-55, Barcelona, Spain) at −60 °C and 0.8 mbar for 72 h. The aerogels obtained were kept in a desiccator with P_2_O_5_ (0% RH) before characterisation. The concentration of total solids in the liquid dispersion and the mass fraction of the different constituents in the solid aerogel are given in Table 1.

### 2.4. Characterisation of Aerogels

#### 2.4.1. Density and Theoretical Porosity

**Figure 1:**
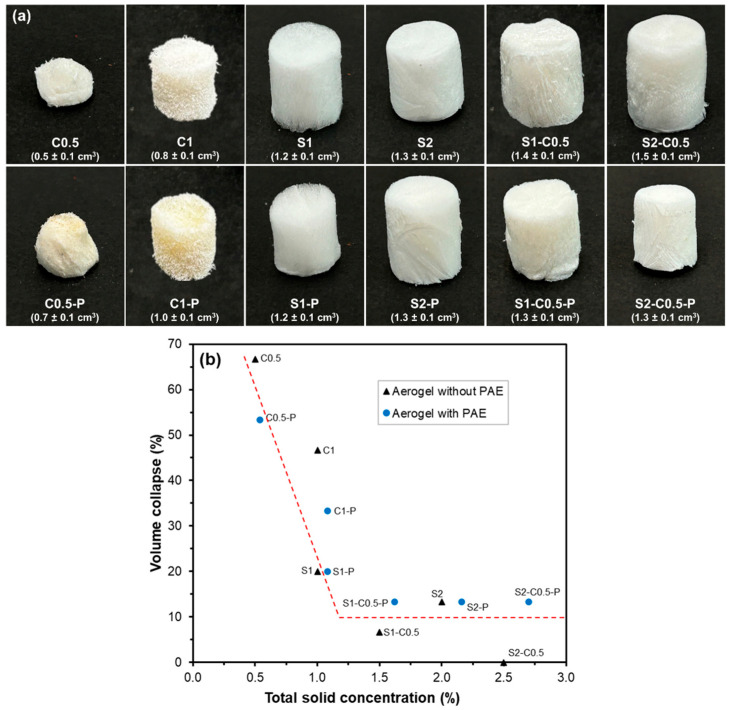
Appearance and volume (cm^3^) (**a**) and correlation between volume collapse vs. total solid concentration of the aqueous dispersion (**b**) for the aerogels obtained with pure cellulose (C0.5 and C1), pure starch (S1 and S2), starch–cellulose blends (S1-C0.5 and S2-C0.5), and the respective samples cross-linked with PAE (-P). The red dash line represents the trend line of volume collapse for aerogels as the concentration of solids increased.

The apparent density of the different aerogels was determined by considering their mass and volume [21]. The mass of the samples was determined with an analytical balance, while their dimensions (Figure 1) were measured with a calliper (Digital calliper, 0–1500 mm) in four different positions for each replicate (six samples per formulation). The theoretical porosity of the aerogels was calculated using Equation (1), where *ρ_a_* is the apparent density of the aerogel and *ρ_x_* is the theoretical density of cellulose (*ρ_C_* = 1676 mg·cm^−3^; Diddens et al. [36]), starch (*ρ_S_* = 1500 mg·cm^−3^; Jane [37]) or those theoretically estimated from the starch–cellulose blends (*ρ_C-S_*) considering the mass fraction of each component (cellulose (*m_C_*) or starch (*m_S_*)) (Equation (2)).

(1) $$Porosity\;\left(\%\right)=\left(1-\frac{\rho_{a}}{\rho_{x}}\right)\times 100\%$$

(2) $$\rho_{C-S}=\frac{1}{\frac{m_{C}}{\rho_{C}}}+\frac{1}{\frac{m_{S}}{\rho_{S}}}$$

#### 2.4.2. Aspect and Microstructure

The internal morphology of the aerogels was evaluated using a High-Resolution Field Emission Scanning Electron Microscope (HRFESEM) (GeminiSEM 500, Zeiss, Oberkochen, Germany). A cylindrical aerogel sample conditioned 0% RH for 1 week at 25 °C, was carefully cross-sectioned using two forceps, and the inner surface was coated with a platinum layer, using an EM MED020 electrospray equipment (Leica BioSystems, Barcelona, Spain) for 60 s. The observations were carried out in the central area of the cylindrical cross-section where the potential impact of the crushing was minimal. The micrographs were taken at 1.5 kV acceleration voltage.

Photographs of the aerogels were taken to compare the appearance, shape, and volume of the samples as a function of their composition.

#### 2.4.3. Thermal Stability

The thermal stability and bound water content of each formulation were determined in duplicate by thermogravimetric analysis (TGA) using a thermogravimetric analyser (TGA 1 Stare System analyser, Mettler-Toledo, Greifensee, Switzerland). Samples of about 3–5 mg were weighed into alumina pans and heated from 25 to 700 °C at a rate of 10 °C·min^−1^ under a constant nitrogen flow (10 mL·min^−1^). The TGA and their derivative (DTGA) curves were analysed to determine the initial (T_on_) and the maximum thermodegradation rate (T_p_) temperatures, as well as the mass loss up to 130 °C (considered as the bound water content).

#### 2.4.4. Sorption Isotherms

Sorption isotherms of the different aerogels were obtained by equilibrating the different samples at different relative humidity, at 25 °C in desiccators containing P_2_O_5_ (0% RH) and different over-saturated salt solutions, typically LiCl, MgCl_2_, K_2_CO_3_, KI, NaCl, KCl, and KNO_3_, with water activities (*a_w_*) of 0, 0.113, 0.3278, 0.4316, 0.6886, 0.7529, 0.8434, and 0.9541, respectively. The samples were conditioned for 30 days and periodically weighed in an analytical balance until a constant weight was reached. Subsequently, the samples were dried in a vacuum oven (0.8 mmbar) at 65 °C for 24 h to determine their equilibrium moisture. The bound water content determined by TGA in dried samples was considered in the total moisture content. The analysis was performed in duplicate for each treatment. The experimental data were fitted to the Guggenheim-Anderson-de Böer (GAB) model (Equation (3)) using OriginPro software (Version 2021, OriginLab Corporation, Northampton, MA, USA).

(3) $$\omega_{e}=\frac{\omega_{0}\cdot C\cdot k\cdot a_{w}}{\left(1-k\cdot a_{w}\right)\cdot \left[1+\left(C-1\right)\cdot k\cdot a_{w}\right]}\;$$

where *ω_e_* is the equilibrium moisture content on a dry basis; *ω*_0_ is the monolayer moisture content; *k* and *C* are equation parameters, which are temperature dependent and related to the water sorption enthalpy; and *a_w_* is the water activity.

In order to determine the specific surface area (SSA = 3.5 × 10^3^ *ω*_0_) of the different aerogels, the BET molecular model was also fitted to the experimental data for *a_w_* < 0.4 to determine the monolayer moisture content (*ω*_0_), as described by Freitas et al. [21].

#### 2.4.5. Mechanical Properties

The mechanical properties of aerogels indicate the durability of the material considering their potential applications. For this analysis, aerogels were submitted to a double compression test, using a mechanical testing machine (TA.XTplus model, Stable Micro Systems, Haslemere, England) equipped with a cylindrical probe (7.5 cm diameter). The samples conditioned at 0% RH at 25 °C for one week were compressed up to a relative strain of 80%, at a strain rate of 3.00 mm·min^−1^, in two cycles, with a recovery time of 30 s [16]. The force–distance curves were used to calculate the compressive stress (*σ*) and the relative Henky strain (*ε_H_*) (Equations (4) and (5)), where *F*(*t*) is the force recorded by the equipment at each time, *L*_0_ (mm) is the initial height of the aerogel, *d*(*t*) is the distance compressed at each time, and *A*_0_ is the initial area of the cylindrical base of the aerogel.

(4) $$\;\sigma \;\left(t\right)=\frac{F(t)}{S(t)}=\frac{F\left(t\right)\cdot (L_{0}+d\left(t\right))}{A_{0}L_{0}}$$

(5) $$\;\varepsilon_{H}=ln\left[\frac{L_{0}+d(t)}{L_{0}}\right]$$

The parameters considered in the *σ* vs. *ε_H_* curve were the stress at the slope change point in the curve (yield stress: *σ_Y_*), the stress at 80% strain (*σ*_80_), and the percentage of height recovered by the sample after the first compression, referred to its initial height (*R*). The analysis was performed in triplicate for each formulation.

#### 2.4.6. Water and Oil Absorption Capacities

The water and oil absorption capacities, as well as the retention capacity of the absorbed liquid, were determined according to Freitas et al. [21] methodology. Briefly, five conditioned aerogels (0% RH, 25 °C for one week) of each formulation were weighed and placed on a grid. The prepared systems were immersed in 30 mL of distilled water or sunflower oil in a Petri dish for 15 min until saturation was achieved. Thereafter, the samples were removed, and the excess liquid trapped in the grid was removed and weighed. The measured water (WAC_M_) and oil (OAC_M_) absorption capacities of the different aerogels were determined according to Equations (6) and (7), respectively, where *w_s_* and *w*_0_ are the masses of the fully water-saturated and dry aerogels, respectively, and the same for oil with *o_s_* and *o*_0_.

(6) $${WAC}_{M}=\frac{w_{s}-w_{0}}{w_{0}}$$

(7) $${OAC}_{M}=\frac{o_{s}-o_{0}}{o_{0}}$$

Theoretical water (WAC_T_) and oil (OAC_T_) absorption capacities were also calculated, considering a constant aerogel volume, the porosity and the densities of aerogel (*ρ_a_*), and water (*ρ_water_* = 0.9982 g·cm^−3^ at 20 °C) or oil (*ρ_oil_* = 0.92 g·cm^−3^ at 20 °C) (Equations (8) and (9)).

(8) $${WAC}_{T}=\frac{Porosity\times \rho_{water}}{\rho_{a}}$$

(9) $${OAC}_{T}=\frac{Porosity\times \rho_{oil}}{\rho_{a}}$$

To determine the water (WRC) and oil (ORC) retention capacity, the saturated samples were placed on absorbent paper and covered with plates to avoid evaporation losses until constant weight was reached. The retention capacities were expressed as the mass of water or oil retained per mass unit of dry aerogel.

### 2.5. Statistical Analysis

The experimental data were analysed through analysis of variance (ANOVA) using a Minitab Statistical Program (version 17) at a confidence level of 95%. To determine whether there were significant differences among the formulations, Tukey’s studentised range (HSD) test was performed, considering a 5% least significant difference (α).

## 3. Results and Discussion

### 3.1. Aspect, Volume Collapse, and Microstructure of the Aerogels

Figure 1 shows the images of the different aerogels, where the average volume of each sample is also shown. All aerogels showed a spongy appearance, with a white colour and homogeneous structure. The aerogels cross-linked with PAE were slightly more yellowish, due to the cross-linking reaction of this polyimide with the other components. The differences in the volume of the aerogels can be mainly attributed to the different total solid concentration in the liquid dispersions, as well as to the different interactions between the components. These factors contribute to the different strength of the three-dimensional network formed during the cryoconcentration of solids in the freezing stage and their rearrangement during the ice sublimation in the freeze-drying step [38]. The interactions between the aerogel components drastically change when the solvent is removed. In the initial hydrogel, water molecules form hydrogen bonds with the polymers, whereas, after sublimation, the strength of the aerogel depends on the hydrogen bonds and other molecular interactions between the polymer chains. Nevertheless, the structuration of the aerogel and its properties are greatly dependent on the initial chain arrangement in the previous hydrogel formed by the cryoconcentration of the polymers during the freezing step [39].

The total solid concentration in the aqueous dispersion greatly affected the final volume of the aerogels up to a critical value, whereas no remarkable differences were observed above this critical concentration, which in the studied systems corresponds to about 1.5%. Dilamian and Noroozi [16] also observed similar behaviour for RS cellulose aerogels, with a critical concentration of about 1.3%. In excessively diluted dispersions, the formation of a cohesive three-dimensional network is compromised by insufficient molecules to form the interlocked network extended to the entire volume of the initial water dispersion (1.5 mL), as it occurs in the water gelification process. Therefore, a greater volume collapse is produced, with respect to the liquid initial volume, when the solid concentration does not reach the critical value. This effect was noticeable in the cellulose aerogels, with and without PAE (C0.5, C0.5-P, C1 and C1-P), where the total solids did not reach the critical value (Figure 1). In contrast, for the formulations with total solids higher than about 1.25%, the volume collapse was much lower and practically constant (about 10%), regardless of the solid composition, as shown in Figure 1. Deviations with respect to the described tendency can be attributed to the different strength of the compounds in the solid aerogel supporting the three-dimensional network. In this sense, pure cellulose aerogels cross-linked with PAE exhibited lower volume shrinkage than those non-cross-linked with similar solid content. In contrast, this effect was not observed for pure starch aerogels, suggesting a low cross-linking effect. The reduction of volume collapse by PAE incorporation in the cellulose aerogels can be attributed to the described cross-linking through the esterification of the azetidinium ions of the PAE chains with carboxyl groups remaining in the cellulose fibres [23,24]. Likewise, the self-cross-linking of PAE chains may promote better structuring of aerogels [23]. Other studies analysed the volume shrinkage of aerogels, reporting 20% for cellulose at 3% wt. [40] or 39–48% in starch–cellulose aerogels with 10–15% wt. total solids [32]. Considering these values, the obtained aerogels at a solid concentration higher than 1.5% exhibited very low volume shrinkage.

**Figure 2:**
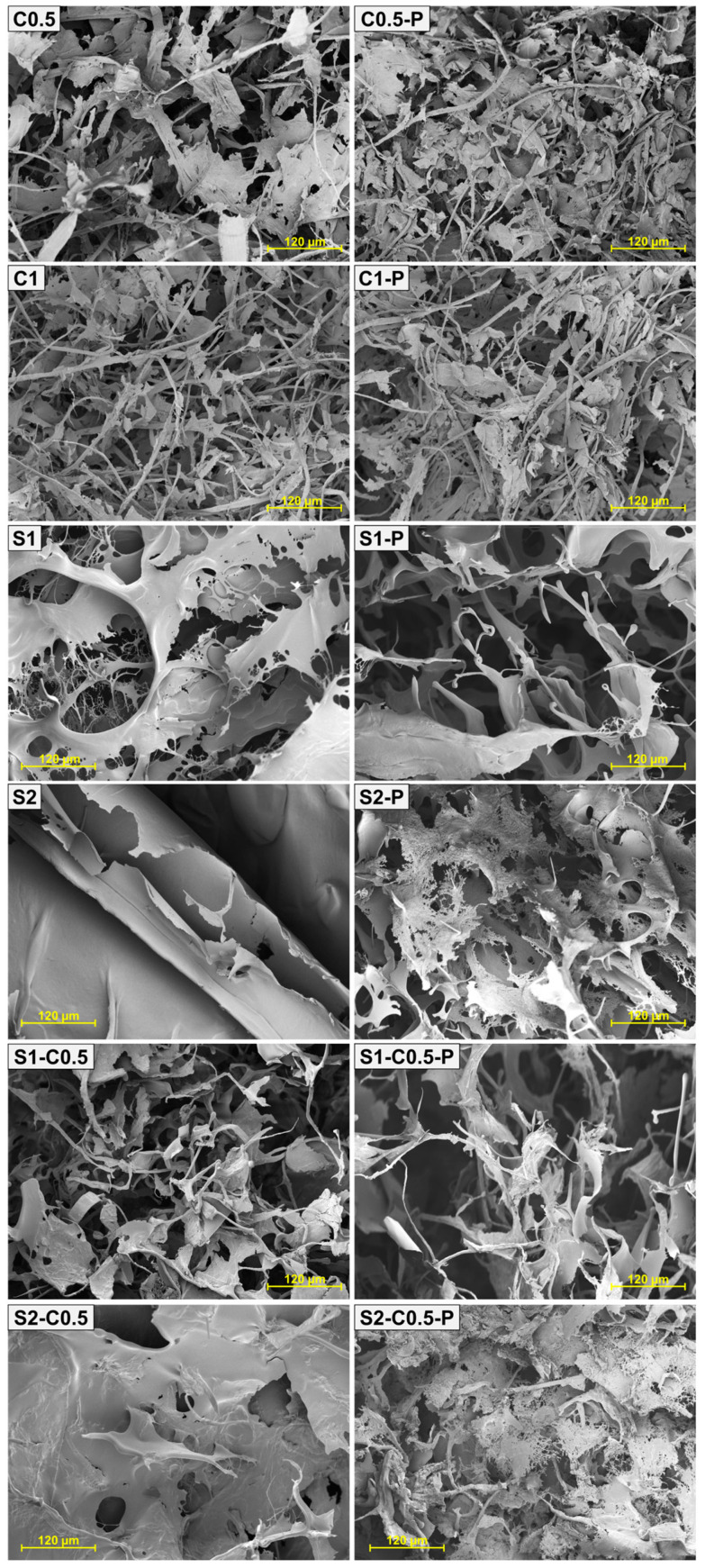
HRFESEM micrographs (200× magnification) of the different aerogels obtained with cellulose (C0.5 and C1), starch (S1 and S2) and cellulose–starch mixtures (S1-C0.5 and S2-C0.5), without and with PAE (-P).

Figure 2 shows the internal microstructure of the aerogels observed by HRFESEM. In general, high porosity and low-density structures are observed, as also reported by other authors [38]. A wide range of pore sizes was noticed, with smaller pores resulting from the interlocking of the polymeric compounds (cellulose and/or starch) and larger pores associated with the voids generated by the ice crystals after sublimation [41]. The higher the cooling rate, the higher the number of nuclei formed and the smaller the crystal sizes, leading to smaller pores in the aerogel structure [21]. Relevant microstructural differences were observed as a function of solid composition. As described by Feng et al. [42], an increase in the cellulose concentration gives rise to a more compact and less porous network. For the pure cellulose aerogels, interconnected fibres and lamellae can be observed, whereas, in pure starch aerogels, only lamellar structure was detected. In the blended cellulose–starch aerogels, the fibrousness increased by the cellulose presence. The lamellae configuration was formed by aggregation of soluble material during the cryo-concentration step of the aqueous dispersion. The lamellar zones coat the cellulose fibrils to a greater extent, as also reported by Luo et al. [33].

The incorporation of PAE implied a slight increase in the solid concentration of dispersion, as well as the potential reinforcing effect, as commented on above. This effect promoted a more compact structure in pure cellulose samples. However, in starch-containing samples, with and without cellulose, the addition of PAE introduced discontinuities in the structure of lamellae, giving them a less compact appearance. This could be attributed to the interruptions in the aggregation zones of starch chains by the presence of the self-cross-linked PAE domains.

### 3.2. Density and Porosity of the Aerogels

**Figure 3:**
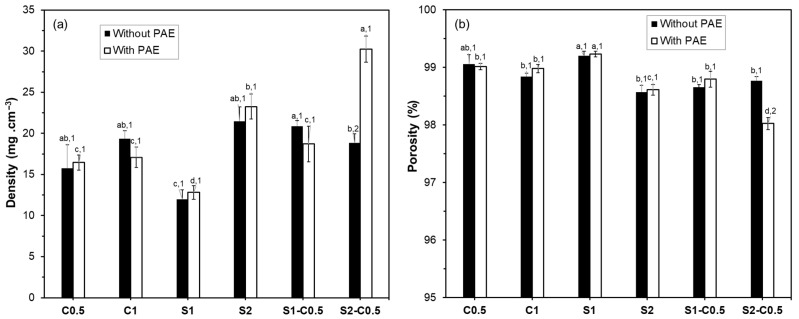
Density and porosity values of the different aerogels obtained with cellulose (C0.5 and C1), starch (S1 and S2), and cellulose–starch mixtures (S1-C0.5 and S2-C0.5), without and with PAE (-P). Different letters indicate significant differences between the samples without PAE or between those containing PAE. Different numbers indicate significant differences between the samples with a determined polymer composition with and without PAE (e.g., C0.5 and C0.5-P).

Figure 3 shows the apparent density and theoretical porosity values of the different aerogels. The density values ranged from 13 to 30 mg·cm^−3^; the S2-C0.5-P sample being the densest, and S1, with and without PAE, presenting the lowest density values. The density and porosity values of the cellulose aerogels were similar to those previously reported for RS cellulose aerogels with similar fibre composition [21] and concentration in the liquid dispersion. However, the higher hemicellulose content in the fibres reduced the density, promoting the porosity of aerogels [21]. Luo et al. [33] obtained cellulose and cellulose–starch aerogels with higher densities (125–151 mg·cm^−3^) and lower porosities (90.1–91.8%). The density and porosity of aerogels were strongly influenced by the solid concentration of the initial dispersion, as already discussed for the volume shrinkage, and by the solid composition. For the same solid concentration in the aqueous dispersion (e.g., 1% wt.), cellulose aerogels were denser and less porous than starch aerogels. Incorporating cellulose into the starch aerogels only provoked a significant increase in density, with the corresponding decrease in porosity, in the S1-C0.5 sample (*p* < 0.05). The non-significant effect of cellulose in the S2-C0.5 aerogel can be attributed to the higher solids’ concentration (above the threshold value) of the initial dispersion. Other studies also reported the effect of the initial solid concentration on the density and porosity of the aerogels [16,38,43]. Moreover, a concentration threshold beyond which further increases in solid content result in negligible changes in the aerogels’ density.

Except for the composite S2-C0.5-P formulation, the incorporation of PAE did not significantly affect the density and porosity of the aerogels, as previously observed by Dilamian and Noroozi [16] for RS cellulose aerogels obtained with different concentrations of solids in the liquid dispersion. However, Nguyen et al. [44] observed that the higher the PAE ratio, the denser, less porous, and stronger the aerogel prepared with microfibrillated cellulose from pineapple leaves. The presence of PAE in the S2-C0.5-P sample resulted in a significantly denser structure, which could be related to the structural effects observed in HRFESEM.

In this aforementioned sense, the effect of solid content on the aerogel formation greatly determines the aggregation degree of constituents, leading to more open (lower solids concentration) or more compact (higher solids concentration) structures. From the threshold concentration, the higher the solids concentration, the greater the number of binding points in the structure, but the air volume within the structure did not change noticeably.

### 3.3. Mechanical Properties of the Aerogels

**Figure 4:**
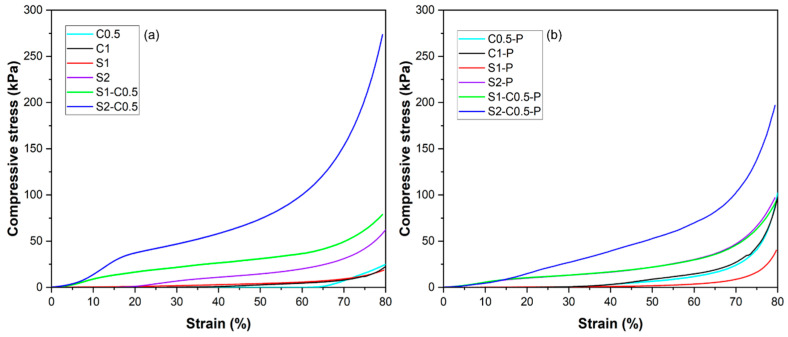
Stress–strain curves obtained at the first compression cycle of the different aerogels prepared with cellulose (C0.5 and C1), starch (S1 and S2), and cellulose–starch mixtures (S1-C0.5 and S2-C0.5) without (**a**) and with PAE (-P) (**b**).

The mechanical resistance of the aerogels was evaluated through a double compression test considering a total deformation of 80% of the initial height of the cylinder. The second compression allows for quantifying the deformation recovered by the structure after the first compression. The stress–strain curves in the first compression cycle for the different formulations are shown in Figure 4, which shows the typical shape for aerogel compression [20]. All specimens presented a quasi-elastic behaviour at low deformations, where the different resistance of the solid network to deformation can be observed. At higher deformations, a change in the slope of the curve occurred, associated with the compression of the porous structure of the material, thus offering a lower resistance to deformation. Finally, as compression progressed, the stress increased exponentially due to the lower proportion of air (densification zone). The mechanical behaviour of aerogels will be affected by the bond strength in the three-dimensional network, the number of bonds per volume unit, and the density or air proportion in the structure.

The mechanical parameters determined for each aerogel, shown in Table 2, were the stress at the first change of slope (yield stress: σ_Y_), the stress at 80% strain (σ_80_), and the percentage of height recovered by the sample after the first compression (R), referred to the initial height. The samples obtained from the low-concentration dispersions (C0.5 and C1 and S1), with lower densities and higher porosities, exhibited the lowest strength values, which can be attributed to the lower bonding points in the network. Likewise, at the same concentration in the initial dispersion, the pure starch aerogels exhibited lower mechanical strength than the cellulose samples but showed the highest percentage of height recovery. This behaviour suggests the existence of stronger bonds in the cellulose network, resulting in a more brittle structure and a greater ability for re-establishing new bonds in the starch network with more flexible behaviour. From 1.5% total solids in the initial dispersion, every sample (S2 and composite aerogels) recovered about 20% of the initial dimension after compression, regardless of the solid composition. However, the composite aerogels exhibited higher strength values than pure starch and pure cellulose samples (300–350% in σ_80_, with respect to the corresponding starch samples without cellulose and 180–842% in σ_80_, with respect to the C0.5 sample). The composite S2-C0.5 samples showed much higher strength than the S1-C0.5 sample (*p* < 0.05), according to the greater ratio of bonding points in the matrix with a higher solid density. This reinforcing effect was not associated with higher volume recovery of the samples, probably due to the lower proportion of air in the structure, which implied greater solid aggregation during compression. Nevertheless, the composite aerogels exhibited higher volume recovery than the pure cellulose aerogels. Zhu et al. [45] also observed that low-density hemp cellulose aerogel (2.1 mg·cm^−3^) recovered more volume after compression than denser aerogels due to the greater interconnection between adjacent fibres that drastically reduced the volume of the compressible air cells.

The incorporation of PAE led to a significant increase in the strength of the pure cellulose aerogels (225 and 280% increase in σ_80_ for C0.5 and C1, respectively), also promoting the volume recovery of the material (43 and 46% increase in R, respectively), as observed by other authors [45]. The reinforcing effect of PAE was also observed in the pure starch samples (111 and 32% increase in σ_80_ for S1 and S2, respectively), but their volume recovery capacity decreased, mainly in the S1 sample. For the mixed samples, PAE slightly increased the strength in the S1-C0.5 aerogel (17% in σ_80_), whereas σ_80_ decreased in the S2-C0.5 sample (25%), probably due to the different starch–cellulose ratio in both samples and the different interaction mechanisms of PAE with each polymer. Therefore, PAE introduced new bonding points in the blended network, modifying the interlacing of the starch and cellulose chains and giving rise to more cohesive but less deformable aerogels.

The PAE cross-linking was especially effective for cellulose aerogels (C0.5, C1) and less effective in starch samples (S1) and composite formulations, especially when the solid concentration of the initial dispersion rose. In these cases, the self-cross-linking of PAE would probably be the more relevant reinforcing mechanism due to the absence of carboxyl groups in starch and their low ratio in the used cellulose, with very low hemicellulose content (~3.4%).

### 3.4. Thermal Stability of the Aerogels

**Figure 5:**
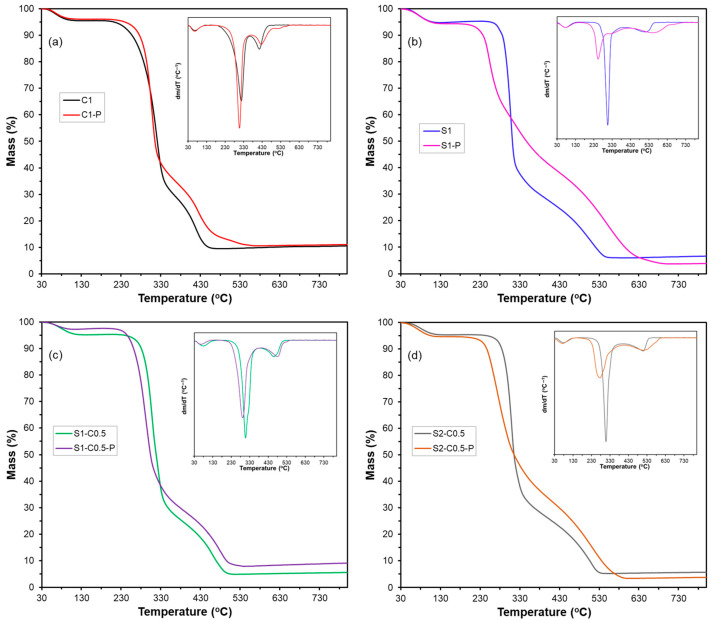
TGA and DTGA (inserted figure) curves of the different aerogels obtained with cellulose (C1) (**a**), starch (S1) (**b**), and cellulose–starch mixtures (S1-C0.5 (**c**) and S2-C0.5 (**d**)) without and with PAE (-P).

The TGA of the samples conditioned at 0% RH allowed for quantifying their bound water content (Table 3) (mass loss at 130 °C), as well as the degradation behaviour of the aerogel polymeric material. Figure 5 shows the TGA and DTGA curves of the different aerogel samples. At about 70 °C (T_on_), a weight loss stage started in all samples that extended up to approximately 130 °C (first event in the DTGA curves). This event corresponds to the loss of bound water from the samples, which is not released under 0% RH conditions due to its strong hydrogen bonding with the polymers. This water content ranged between 2.5 and 6.5%, depending on the sample, being lower in the samples with PAE (Table 3).

The decomposition of the polymeric material occurred between 220 and 330 °C, with a mass loss of more than 50%. This event is associated with the breakdown of the cellulose or starch structure into volatile compounds and CO_2_, as observed by other authors [46]. The temperatures of maximum degradation rate (T_p_) (second peak in the DGTA curve) agree with those reported for cellulose (330 °C, Freitas et al. [7]) and starch (300 °C, Liu et al. [47]). Therefore, the degradation of both polymers overlaps in the mixed aerogels. The polymer decomposition products and residual lignin of cellulose degrade at higher temperatures, between 400 and 550 °C. Above 550 °C, the residual mass corresponds to the ash and carbon material resulting from the pyrolysis of the samples [20].

The incorporation of PAE modified the thermodegradation behaviour of the aerogels, especially for the starch-containing samples. Although there was no noticeable change in the pattern of cellulose degradation, the starch aerogels exhibited a significant temperature shift toward lower values (~240 °C), with the secondary degradation peaks at higher temperatures. This effect was more evident in the pure starch aerogels, which suggests that the interactions between PAE and starch chains are responsible for the lower thermal stability of the samples. These interactions were already deduced from the micrographs of the aerogels with PAE (Figure 2), where the aerogel lamellae in starch cross-linked showed a porous structure. This could indicate that PAE disrupts the starch chain associations in the aerogel, thereby limiting their level of aggregation. In fact, the thermal stability of the material is strongly affected by its degree of compactness and crystallinity.

Therefore, the thermal analysis reveals the weakening of the starch structure in the aerogel by the PAE incorporation, and so, the observed mild reinforcing effect can be mainly attributed to the PAE self-cross-linking. The self-cross-linked PAE network provides the aerogels with higher strength, but the cooperative effect with both polymers (cellulose and starch) differed. The PAE network did not affect the chain arrangement for the cellulose formulations, but it noticeably affected the starch network.

### 3.5. Water Sorption Isotherms of the Aerogels

**Figure 6:**
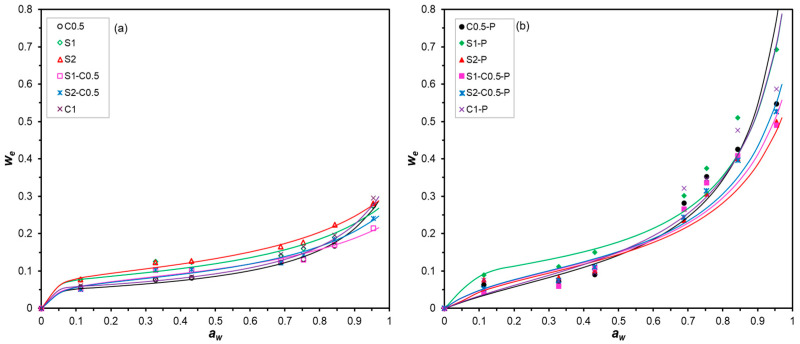
Sorption isotherms (experimental points and fitted GAB model: curves) of the different aerogels obtained with cellulose (C0.5 and C1), starch (S1 and S2), and cellulose–starch mixtures (S1-C0.5 and S2-C0.5) with and without PAE (-P).

Figure 6 shows the experimental points (equilibrium moisture content vs. a_w_) and the fitted GAB model for the different aerogels. Likewise, Table 3 gathers the parameters of the GAB fitted model, the specific surface area (SSA, deduced from the BET monolayer moisture content), and the bound water content for each formulation. All aerogels showed type II isotherms, according to the IUPAC classification, which is characteristic of solids with a specific surface area for the adsorption of a monolayer of water molecules in active points at low relative humidity (a_w_ < 0.6), and multilayer adsorption at high relative humidity (a_w_ > 0.6) [46]. In porous systems, capillary water condensation can occur when the water vapour pressure in the pore is reached as a function of its radius. Differences in water sorption behaviour between the different aerogels are attributed to the different material compositions and may also be affected by their porosity.

Pure starch aerogels exhibited higher water sorption capacity than pure cellulose aerogels, the mixed samples exhibiting an intermediate behaviour. The strong aggregation of cellulose fibres through hydrogen bonds results in fewer active sites for water adsorption than in the starch formulations. The presence of PAE affected the water adsorption capacity of the aerogels. At low a_w_ (<0.6), the equilibrium moisture values of the samples cross-linked with PAE were slightly lower than those of the aerogels without PAE. This indicates the blocking of active sites for water adsorption by the cross-linking effect. This result was coherent with the lower values of bound water content determined by TGA in samples with PAE (Table 3). Nevertheless, from a_w_ > 0.6, all aerogels with PAE exhibited a more intense increase in equilibrium moisture than that observed for samples without PAE. This suggests that capillary condensation of water molecules occurs in the smaller pores of the cross-linked structure. These small pores could collapse in the non-cross-linked aerogels due to the water adsorption itself, which breaks the interlocking of the polymers in the dry aerogel structure, by forming new hydrogen bonds with water molecules. Therefore, PAE reinforced the structure of aerogels, limiting their collapse under high relative humidity conditions while enhancing their water sorption capacity. This effect was especially favoured in the aerogels with higher porosity, with a lower solid concentration in the initial aqueous dispersion (C0.5-P, C1-P and S1-P).

The specific surface area (SSA) of aerogels (Table 3), determined from the BET monolayer moisture content, ranged between 163 and 303 m^2^·g^−1^. The cellulose aerogels showed the lowest values of SSA, coherently with the lower proportion of adsorption active sites due to the higher proportion of interchain bonds. The presence of cellulose in the composite aerogels also decreased the SSA values. This effect was also observed by Dogenski et al. [32] for composite aerogels, with SSA values between 65 and 190 m^2^·g^−1^. Likewise, Druel et al. [25] reported that as the amylose content of starch aerogels increased, the specific surface area (SSA) increased, reflecting the relevant role of amylose in the aerogel structuring. These authors reported values of SSA of 90 m^2^·g^−1^ for the waxy potato starch aerogel, while in the high amylose corn starch aerogel SSA was 250 m^2^·g^−1^. A slight increase in the SSA values was observed for the cross-linked aerogels, except for S2 samples, which also reveals the enhancing effect of PAE on the water adsorption capacity of the samples, both at monolayer level by increasing SSA and by capillary condensation.

### 3.6. WAC and OAC of the Aerogels

**Figure 7:**
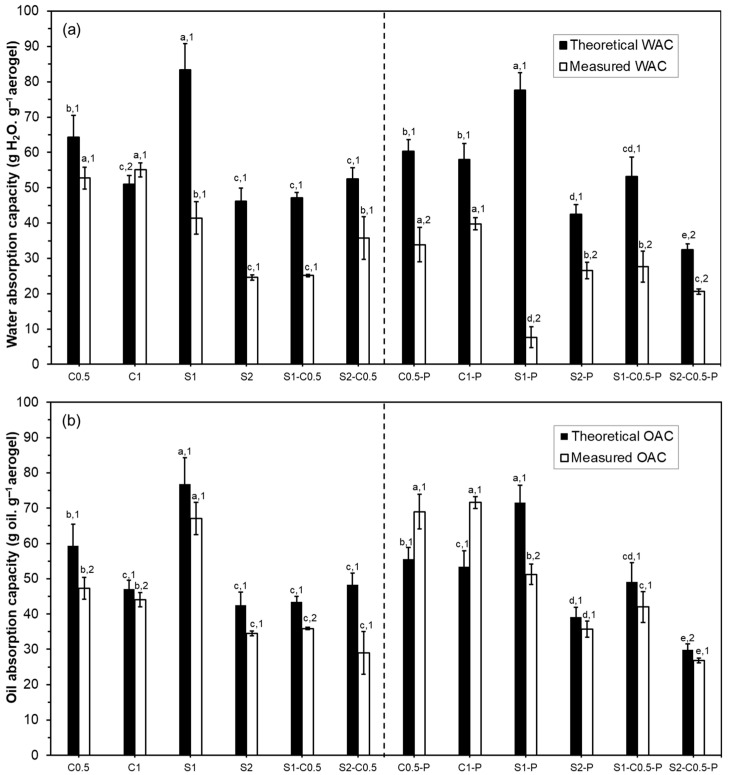
Theoretical (black bars) and experimental (white bars) water (WAC) (**a**) and oil (OAC) (**b**) absorption capacities of the different aerogels obtained with cellulose (C0.5 and C1), starch (S1 and S2), and cellulose–starch mixtures (S1-C0.5 and S2-C0.5), with and without PAE (-P).

The theoretical (subscript T) and measured (subscript M) water (WAC) and oil (OAC) absorption capacities of the aerogels are shown in Figure 7. Except for the C1 aerogel, the WAC_M_ values were lower than their respective theoretical values (WAC_T_). These differences are attributable to the partial collapse of the aerogel structure in contact with the liquid water. The water contact reduced the binding forces of the three-dimensional network due to the partial solubility of the components and the water ability to break hydrogen bonds in the polymeric matrix, forming new water–polymer bonds. The smaller difference between the WAC_T_ and WAC_M_ values in the C1 sample reveals that its structure does not collapse in contact with liquid water, and even the partial swelling of the network. In general, pure cellulose aerogels (less water soluble) showed smaller differences between WAC_T_ and WAC_M_ than pure starch aerogels (more water soluble) and composite samples, which exhibited intermediate differences. The increase in the solid concentration of the liquid dispersion also reduced these differences. In line with this, the experimental values of WAC for cellulose aerogels were higher than for starch aerogels and composite formulations. So, the water solubility of the polymer and the number of binding points in the matrix greatly affected the water absorption capacity of the aerogels. Fontes-Candia et al. [48] reported WAC values ranging between 20 and 50 g·water·g^−1^ aerogel, using cellulose produced from Arundo donax waste with different purification degrees, which are in the range of the obtained values. The C0.5 sample showed similar values to that reported for aerogels obtained at the same concentration with RS cellulose fibres with similar composition [21].

The cross-linking with PAE did not improve the WAC of cellulose aerogels while increasing the differences between WAC_T_ and WAC_M_. However, Tetik et al. [49] reported a lower disintegration of cellulose aerogels cross-linked with PAE when immersed in liquid water. The cross-linking could reduce the swelling capacity of the cellulose aerogels, limiting their absorption capacity. The effect of PAE in starch-containing aerogels was greatly negative on the WAC values of the S1 samples while also reducing the WAC of the other starch formulations. This could be related to promoting the starch phase solubilisation in the more porous structure of the aerogel lamellae observed by HRFESEM (Figure 2).

As expected, differences between OAC_T_ and OAC_M_ values were lower than for WAC_T_ and WAC_M_, since triglycerides have lower chemical affinity and interaction capacity with the aerogel components, giving rise to lower structural collapse during oil immersion. The starch aerogels exhibited higher oil absorption capacity for the same solid concentration than cellulose aerogels, although cellulose aerogels also showed very low differences between OAC_T_ and OAC_M_. Paulauskiene et al. [35] reported an increase in the oil absorption capacity in composite cellulose–starch aerogels with respect to cellulose aerogels. The S2C0.5 formulation exhibited the highest difference between OAC_T_ and OAC_M_, and the lowest oil absorption capacity, suggesting a greater collapse of the structure provoked by oil. Fontes-Candia et al. [48] reported OAC values of cellulose aerogels in the same range (between 30 and 60 g·oil·g^−1^ sample) while observing an increase in OAC when the cellulose purity increases in the aerogels produced from Arundo donax. Therefore, the composition of the aerogels greatly affects both water and oil absorption capacity.

The cross-linking with PAE improved the OAC of the cellulose aerogels, even promoting the swelling of the structure (OAC_T_ < OAC_M_), but reduced this capacity in starch aerogels with 1% solids, without notable effect on the other formulations. This suggests that PAE promoted the oil interaction capacity of the cellulose aerogels, enhancing the absorption into the three-dimensional structure while negatively affecting the oil absorption capacity of starch. Therefore, cross-linking with PAE did not notably improve the water absorption capacity of the cellulose aerogels but provided them with excellent oil absorption properties.

The water and oil retention capacity of the different aerogels are also shown in Table 4. The values of WRC ranged between 6 and 11 g·water·g^−1^ aerogel, the S2 sample exhibited the greatest values and the S1C0.5 the lowest one. The incorporation of PAE did not significantly change the WRC of cellulose aerogels but highly reduced this capacity to near 0 in starch-containing samples. In general, the ORC values were greater than WRC values (except for the S2 sample), with the S1 sample showing the highest values and S2 the lowest, without significant differences between the other samples. The incorporation of PAE also slightly improved the ORC of cellulose aerogels but reduced or not the ORC of starch-containing aerogels.

## 4. Conclusions

It was possible to obtain cellulose and starch–cellulose composite aerogels, using RS green cellulose obtained with a more environmentally friendly process. The effect of the aerogel cross-linking with PAE was also analysed. The properties of the cellulose aerogels were in the range of those reported using other RS cellulose fibres with similar compositions. Blending the cellulose with starch implied an increase in mechanical strength and flexibility, compared to pure cellulose aerogels, as well as in oil absorption capacity, while the water absorption capacity decreased. PAE incorporation promoted the water adsorption capacity of all aerogels and the oil absorption capacity and mechanical strength of cellulose aerogels but did not benefit the properties of cellulose–starch composites or pure starch areoles due to specific interactions with starch that negatively affect the aerogel structure. Therefore, the present study demonstrated the possibility of producing aerogels from RS cellulose obtained by a more sustainable method for different applications, modulating their properties by adding starch and cross-linking agents, such as PAE. Considering the properties of the obtained aerogels, these could be used as oil absorbers, especially those of pure cellulose with PAE that exhibited the maximum oil absorption capacity.

## Figures and Tables

**Table 1:** Total concentration of solids in the liquid dispersions (C, g/100 g) and mass fraction of the components (cellulose, starch, and PAE) in the solid phase of the different aerogels.

Formulation	C	Cellulose	Starch	PAE
C0.5	0.50	1.00	0.00	0.00
C1	1.00	1.00	0.00	0.00
S1	1.00	0.00	1.00	0.00
S2	2.00	0.00	1.00	0.00
S1-C	1.50	0.33	0.67	0.00
S2-C	2.50	0.20	0.80	0.00
C0.5-P	0.54	0.93	0.00	0.07
C1-P	1.08	0.93	0.00	0.07
S1-P	1.08	0.00	0.93	0.07
S2-P	2.16	0.00	0.93	0.07
S1-C0.5-P	1.62	0.31	0.62	0.07
S2-C0.5-P	2.70	0.19	0.74	0.07

**Table 2:** Mechanical parameters of the different aerogels: stress at the inflexion point of the curve (σ_Y_), at 80% compression (σ_80_), and recovery of height with respect to the initial height (R).

Formulation	σ_Y_ (kPa) *	σ_80_ (kPa) *	R (%) *
C0.5	2.3 ± 0.6 ^c,2^	28 ± 2 ^d,2^	8.9 ± 0.1 ^c,2^
C1	3.5 ± 0.8 ^c,2^	25 ± 3 ^d,2^	11.0 ± 2.0 ^c,2^
S1	1.4 ± 0.2 ^d,1^	17 ± 3 ^e,2^	40.0 ± 5.0 ^a,1^
S2	11.0 ± 2.0 ^b,1^	66 ± 6 ^c,2^	21.0 ± 3.0 ^b,1^
S1-C0.5	11.0 ± 2.0 ^b,1^	78 ± 1 ^b,2^	20.0 ± 3.0 ^b,2^
S2-C0.5	33.0 ± 1.0 ^a,1^	264 ± 1 ^a,1^	21.0 ± 4.0 ^b,1^
C0.5-P	3.6 + 0.1 ^c,1^	91 ± 10 ^b,1^	12.8 ± 1.0 ^d,1^
C1-P	9.0 + 0.7 ^b,1^	95 + 7 ^b,1^	16.1 + 0.5 ^c,1^
S1-P	0.9 ± 0.1 ^d,1^	36 ± 5 ^c,1^	16.0± 0.7 ^c,2^
S2-P	7.4 ± 1.5 ^b,1^	87 ± 14 ^b,1^	19.3 ± 1.0 ^b,1^
S1-C0.5-P	8.6 ± 1.6 ^b,1^	91 ± 4 ^b,1^	26.6 ± 1.6 ^a,1^
S2-C0.5-P	25.0 ± 2.0 ^a,2^	198 ± 2 ^a,2^	23.0 ± 2.0 ^a,1^

* Different subscript letters in the same column indicate significant differences between samples of the same group (formulations without or with PAE). Different numbers indicate significant differences between aerogels of the same composition without or with PAE (Tukey’s test, *p* < 0.05).

**Table 3:** Bound water, fitted GAB model parameters, and specific surface area (SSA) values (m^2^·g^−1^) obtained from BET analyses of sorption data for a_w_ < 0.4 for the different aerogels.

Formulation	Bound Water (%) *	GAB				BET
		*ω*_0_	*C*	*K*	*R* ^2^	SSA
C0.5	5.0 ± 2.1	0.049	269.1	0.854	0.991	163
C1	4.5 ± 0.7	0.052	225.1	0.788	0.991	178
S1	6.0 ± 0.5	0.077	139.8	0.738	0.998	259
S2	6.5 ± 1.4	0.088	67.2	0.719	0.992	267
S1-C	4.7 ± 0.1	0.072	36.7	0.691	0.995	240
S2-C	4.6 ± 0.2	0.067	43.9	0.754	0.998	257
C0.5-P	2.5 ± 0.3	0.072	3.9	0.918	0.939	173
C1-P	3.9 ± 0.5	0.081	4.5	0.812	0.951	185
S1-P	5.6 ± 0.1	0.104	58.7	0.900	0.941	303
S2-P	3.9 ± 0.7	0.092	7.3	0.835	0.918	194
S1-C0.5-P	2.6 ± 0.8	0.083	8.3	0.851	0.907	245
S2-C0.5-P	5.4 ± 0.2	0.084	8.4	0.863	0.962	257

* Bound water content determined by TGA.

**Table 4:** Water (WRC) and oil (ORC) retention capacities of the different aerogels obtained with cellulose (C0.5 and C1), starch (S1 and S2), and cellulose–starch mixtures (S1-C0.5 and S2-C0.5), with and without PAE (-P).

Formulation	WRC * (g·Water·g^−1^ Aerogel)		ORC * (g·Oil·g^−1^ Aerogel)	
	Without PAE	With PAE	Without PAE	With PAE
C0.5	9.9 ± 4.4 ^ab,1^	11.6 ± 3.8 ^a,1^	14.5 ± 1.3 ^b,2^	19.9 ± 2.2 ^a,1^
C1	9.4 ± 1.2 ^ab,1^	9.6 ± 5.3 ^a,1^	11.3 ± 1.1 ^b,2^	16.0 ± 2.5 ^a,1^
S1	7.5 ± 1.1 ^b,1^	0.2 ± 0.1 ^b,2^	17.1 ± 3.1 ^a,1^	5.5 ± 3.5 ^b,2^
S2	11.0 ± 1.7 ^a,1^	0.1 ± 0.1 ^b,2^	7.7 ± 2.9 ^c,1^	7.8 ± 2.3 ^b,1^
S1-C0.5	5.7 ± 0.9 ^b,1^	0.4 ± 0.3 ^b,2^	11.5 ± 0.9 ^bc,1^	8.5 ± 3.1 ^b,1^
S2-C1	6.7 ±1.2 ^b,1^	0.8 ± 0.5 ^b,2^	14.0 ± 1.9 ^b,1^	6.7 ± 1.1 ^b,2^

* Different subscript letters in the same column indicate significant differences between samples of the same group (formulations without or with PAE). Different numbers in the same line indicate significant differences between aerogels of the same composition without or with PAE (Tukey’s test, *p* < 0.05).

## Data Availability

Data are contained within this article.
